# Effectiveness of Minitracheostomy After Extubation in Patients with Pneumonia at High Risk of Reintubation: A Case Series

**DOI:** 10.2478/jccm-2023-0029

**Published:** 2023-11-14

**Authors:** Akira Ouchi, Yuji Takahashi, Hidehiko Nakano, Masaki Mochizuki, Saiko Okamoto, Hideaki Sakuramoto, Kensuke Nakamura

**Affiliations:** Ibaraki Christian University, Ibaraki, Japan; Hitachi General Hospital, Hitachi, Japan; Japanese Red Cross Kyushu International College of Nursing, Kyushu, Japan

**Keywords:** airway extubation, ventilator weaning, tracheostomy, sputum, pneumonia

## Abstract

**Introduction:**

Minitracheostomy involves the percutaneous insertion of a 4-mm-diameter cricothyroidotomy tube for tracheal suctioning to facilitate the clearance of airway secretions. The advantage of using the minitracheostomy is in the clearance of secretions, however data on their usefulness for respiratory failure after extubation is limited. Aim of the study: We aimed to assess the use of minitracheostomy for patients with challenging extubation caused by significant sputum.

**Material and Methods:**

We conducted a retrospective analysis of consecutive case series. We analyzed the data of 31 patients with pneumonia. After minitracheostomy, the primary endpoints of reintubation within 72 hours and clinical effects, including mortality, length of intensive care unit (ICU), or hospital stay, were assessed. The successful extubation group included patients who did not require reintubation within 72 hours. Conversely, the reintubation group consisted of patients mandating reestablishment of intubation within 72 hours.

**Results:**

Among those who underwent minitracheostomy after extubation, 22 (71%) underwent successful extubation and 9 underwent reintubation (reintubation rate: 29%). The in-hospital mortality rates after 30 days were 18.2% in the successful extubation group and 22.2% in the reintubation group. The ICU and hospital lengths of stay were 11 days (interquartile range: 8–14.3 days) and 23 days (interquartile range: 15.5–41 days), respectively, in the successful extubation group; they were 14 days (interquartile range: 11–18.5 days) and 30 days (interquartile range: 16–45.5 days), respectively, in the reintubation group.

**Conclusions:**

The prophylactic use of minitracheostomy may be an option as a means of reducing reintubation in patients with pneumonia who are at very high risk of reintubation.

## Introduction

Post-extubation management is essential for mechanically ventilated patients. In the intensive care unit (ICU), up to 35% of patients will require reintubation within 48–72 h after planned extubation [1]. Reintubation has been associated with prolonged mechanical ventilation, extended ICU and hospital stays, and increased mortality [2,3]. Therefore, extra-precaution is required before performing extubation to prevent reintubation.

Minitracheostomy is a modified tracheostomy procedure involving the percutaneous insertion of a 4-mm-diameter cricothyroidotomy tube for suctioning to facilitate the clearance of airway secretions [4]. The biggest advantage is that it is less invasive than a tracheostomy, and once the tube is removed the wound closes faster than a tracheostomy wound. In addition, medical personnel can suction secretion around the carina through the tube as a temporary access point. Minitracheostomy is commonly used to assist in the management of copious secretion retention.

A common clinical rationale for the insertion of a minitracheostomy is to assist in managing copious sputum retention. Literature has described the application of this airway in some surgical patients with this postoperative pulmonary complication [5,6]. A systematic review of 6 studies showed that minitracheostomy facilitates sputum clearance in patients who have undergone surgery or have an acute condition following sputum retention [7]. However, there are few reports on its effectiveness after ventilator weaning, and its efficacy against respiratory failure after extubation is unknown.

In our hospital, minitracheostomy has been used for patients with copious amounts of sputum and challenges during extubation. To our knowledge, no clinical study to date has investigated the outcome of this procedure for ventilator weaning among patients with pneumonia. Therefore, this study aimed to describe the characteristics and clinical outcomes of a consecutive series of extubated patients with pneumonia who underwent prophylactic minitracheostomy.

## Materials and Methods

### Study design

We conducted a retrospective analysis of consecutive case series. We retrospectively reviewed the clinical records of all the hospitalized patients with pneumonia who underwent minitracheostomy (Mini-Trach II; Smiths Medical, Plymouth, MN, USA) after extubation in the Department of Emergency and Critical Care Medicine Hitachi General Hospital. A series of 31 patients with pneumonia were selected from among 46 patients who underwent minitracheostomy at our institution between April 2013 and December 2017.

### Ethical considerations

The procedures were in accordance with the ethical standards of the responsible committee on human experimentation and with the Helsinki Declaration of 1975, as revised in 2000. This retrospective study was approved by the Hitachi General Hospital Institutional Review Board (2017–95). Informed consent for performing minitracheostomy was obtained from the patients or their family members.

### Inclusion and exclusion criteria

The inclusion criteria were minitracheostomy tube insertion within 24 h post-extubation, follow-up ventilatory management for pneumonia, and prophylactic minitracheostomy use. The exclusion criteria were the use of minitracheostomy to treat acute airway obstruction, use of a device other than Mini-Trach II (Smiths Medical), and requirement for ventilatory management of diseases other than pneumonia.

Pneumonia is diagnosed based on chest X-ray findings of infiltration and at least two of the following: fever (>38°C); leukocytosis or leukopenia; and purulent sputum [8]. Extubation criteria were (1) resolution or improvement of the condition that led to intubation, (2) hemodynamic stability, (3) a score of 13 or higher on the Glasgow Coma Scale, and (4) respiratory stability.

Minitracheostomy was indicated post-extubation for cases with adequate oxygenation, maintained ventilation, and stable respiratory frequency, but with anticipated difficulty during extubation due to a large amount of sputum, such as 1-time suction per hour.

### Minitracheostomy procedure

**Fig. 1.**
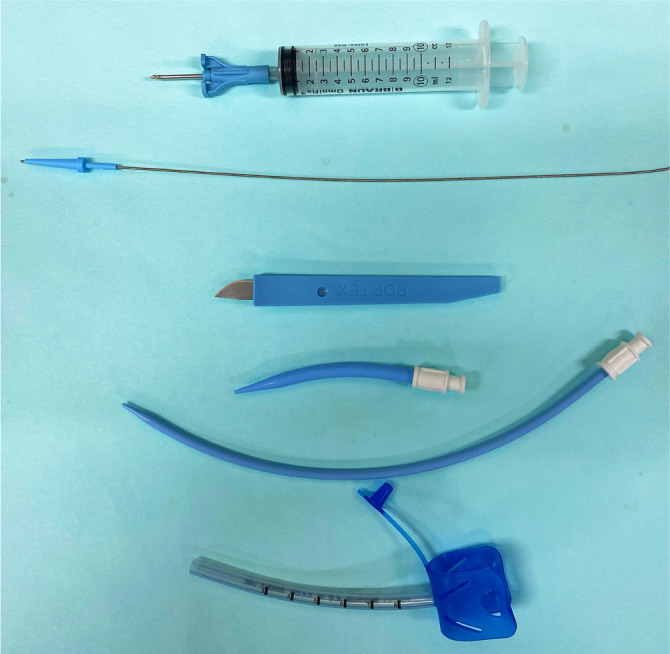
The minitracheostomy kit.

We performed minitracheostomy as shown in Figure 1. The patient was positioned in supine with the neck extended over a pillow. The Seldinger method was used to insert the minitracheostomy tube. The cricothyroid membrane was punctured using a 16-gauge needle. On aspiration of air to confirm the tracheal position of the needle, a guidewire was inserted through the needle and dilated. After removing the needle, a specifically designed endotracheal tube was introduced into the tracheal lumen over the guidewire; then, the guidewire was removed.

Secretions were aspirated using a 10-Fr aspiration cannula via the inserted tube as necessary. When the amount of secretions decreased and cannula aspiration was no longer needed, the minitracheostomy tube was removed and the wound closed spontaneously.

### Data collection and outcome measures

Data regarding age; sex; acute physiology and chronic health evaluation (APACHE) II score; sequential organ failure assessment (SOFA) score; confusion (Glasgow coma scale < 15), uremia (blood urea nitrogen > 20 mg/dL), elevated respiratory rate (respiratory rate 30 breaths/min), hypotension (systolic < 90 mmHg or diastolic 60 mmHg), and age >65 years (CURB-65) score for pneumonia severity; and frequency of suctions performed within 24 h before extubation were obtained from the patients’ records.

The primary endpoints were the proportion of patients who required reintubation within 72 h. The secondary outcome was the clinical effects, including mortality, length of ICU and hospital stays, and tracheostomy rate, for the successful extubation and reintubation groups. The successful extubation group was defined as patients who were not reintubated within 72 hours, and the reintubation group was defined as patients mandating reestablishment of intubation within 72 hours.

Reintubation was considered when the following signs of respiratory failure were noted: respiratory acidosis with pH lower than 7.30 and PaCO_2_ (partial pressure of carbon dioxide) higher than 45 mmHg, oxygen saturation less than 90% or PaO_2_ less than 60 mmHg with FIO_2_ (fraction of inspired oxygen) higher than 0.5, respiratory rate higher than 35 breaths/min, low level of consciousness (Glasgow Coma Scale score <13), severe agitation, some pulmonary x-ray findings (atelectasis, pulmonary consolidation, etc), or clinical signs of respiratory fatigue. Finally, reintubation after extubation was left to the discretion of the attending physician.

### Data analysis

Data were summarized using Excel (Microsoft; Redmond, WA, USA) and are presented descriptively as numbers and percentages for nominal data and as medians and interquartile ranges (IQRs) for continuous data. However, no statistical tests were performed because of the small sample size.

## Results

### Characteristics

**Fig. 2:**
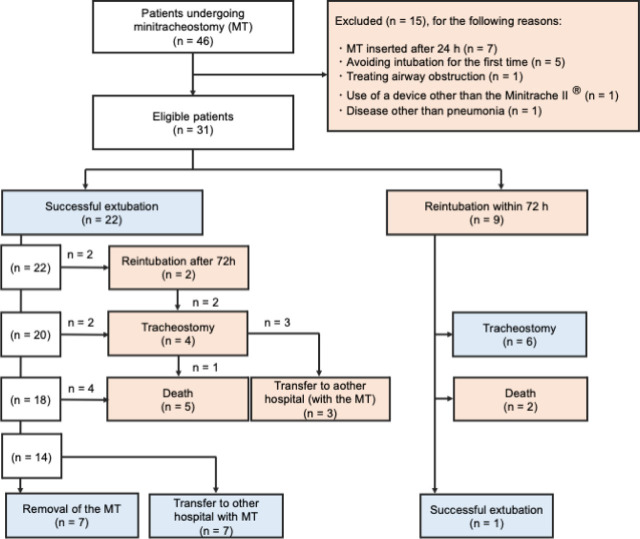
Flowchart of patient inclusion in the study. Thirty-one patients with pneumonia were selected from 46 patients who underwent minitracheostomy at our institution between April 2013 and December 2017. Ten patients required tracheostomy. Eight patients did not require intubation, tracheostomy, or minitracheostomy.

A series of 31 patients with pneumonia were selected. Flowchart of patient inclusion in the study were shown in Figure 2. The characteristics of the patients included in this study are shown in Table 1. The median (IQR) age was 79 (67–84) years. The oldest patient was 92 years old. There were 25 males and 6 female patients. The median (IQR) APACHE II, SOFA, and CURB-65 scores were 19 (17–24), 9 (7–11), and 3 (2–4), respectively. Among the patients who underwent mini-tracheostomy after extubation, 22 (71%) experienced successful extubation and 9 required reintubation. The reintubation rate was 29%. The median (IQR) lengths of mechanical ventilation were 6 (4–10) days in the successful extubation group and 7 (5.5–10) days in the reintubation group. There was no apparent difference in the background data of the patients in the successful and reintubation groups. In particular, the numbers of suctions performed within 24 h before extubation were similar.

**Table 1.** Demographic and clinical characteristics of the patients

	**Overall n=31**	**Successful extubation n=22**	**Reintubation n=9**
Age, years, median (IQR)	79 (67–84)	76.5 (64.8–84.5)	80 (71.5–85)
Male, n(%)	25 (80.6)	21 (95.5)	4 (44.4)
APACHE II score, median (IQR)	19 (17–24)	18 (16–23.3)	19 (18–28)
SOFA score, median (IQR)	9 (7–11)	9.5 (7–11.3)	7 (5.5–11)
CURB-65 score, median (IQR)	3 (2–4)	3 (3–4)	3 (2–3.5)
MV duration, median (IQR)	6 (5–10)	6 (4–10)	7 (5.5–10)
Suctions within 24 hours before extubation, median (IQR)	10 (7–12)	10 (7.8–12)	10 (6.5–12)

IQR, interquartile range; APACHE II, acute physiology and chronic health evaluation II; SOFA, sequential organ failure assessment; CURB-65, confusion, uremia, elevated respiratory rate, hypotension, and age >65; MV, mechanical ventilation

### Clinical outcomes

The clinical outcomes are shown in Table 2. No blood gas was obtained prior to or following the minitracheostomy procedure. Minitracheostomy was successfully and un eventfully performed in all cases, with no observed alterations in SpO2 levels pre and post intervention. The reintubation rate within 72 h was 29%. The 30-day in-hospital mortality rates were 18.2% in the successful extubation group and 22.2% in the reintubation group. The median (IQR) ICU and hospital lengths of stay were 11 (8–14.3) days and 23 (15.5–41) days, respectively, in the successful extubation group, and 14 (11–18.5) days and 30 (16–45.5) days, respectively, in the reintubation group. The most common reason for reintubation was sputum blockage (89%). Some patients had worse outcomes after reintubation. In the successful extubation group, the minitracheostomy tube was removed from seven patients during hospitalization, and seven patients were transferred to another hospital with the minitracheostomy tube in place. Only two patients required reintubation after 72 h (2/22; 9%). In the reintubation group, one patient (1/9; 11%) was successfully re-extubated. Relevant outcomes of the nine reintubated patients included discharge-intubated tracheostomy (6/9; 66.7%). In contrast, 4 of 22 (18.2%) patients needed tracheostomy in the successful extubation group. Overall, 10 patients (32.3%) underwent tracheostomy.

**Table 2.** Patient outcomes

	**Overall n=31**	**Successful extubation n=22**	**Reintubation n=9**
30-day mortality, n (%)	6 (19.4)	4 (18.2)	2 (22.2)
ICU length of stay, days, median (IQR)	11 (9–15)	11 (8–14.3)	14 (11–18.5)
Hospital length of stay, days, median (IQR)	26 (16–40)	23 (15.5–41)	30 (16–45.5)
Tracheostomy, n (%)	10 (32.3)	4 (18.2)	6 (66.7)

ICU, intensive care unit; IQR, interquartile range

## Discussion

In this retrospective analysis of cases that underwent minitracheostomy for extubation, approximately 29% of patients at high risk of reintubation were reintubated, while 71% were successfully extubated.

This study to describe the patient characteristics and outcome of prophylactic use of minitracheostomy in post-extubated patients. The reintubation rate within 72 h was 29%, which was better than that observed during a previous study of common ventilator patients [1]. Also, the reintubation rate was better than the extubation failure rate for patients with weak cough who required frequent endotracheal suctioning [9]. Our results suggest useful features of minitracheostomy because it was expected that the reintubation rate would be higher since only high-risk patients who were likely to have failed extubation were included. Therefore, the prophylactic use of minitracheostomy may be an option as a means of reducing reintubation in patients with pneumonia who are at very high risk of reintubation.

Previous studies have reported tracheostomy rates of 10% to 15% in the intensive care unit [10]. Our findings show that a notably higher tracheostomy requirement among patients, reaching 32.3%, a figure warranting concern. Older age and high reintubation rates may have been factors contributing to prolonged ventilation, and the inclusion of older individuals with pneumonia possibly contributed to prolonged mechanical ventilation and tracheostomy.

To prevent extubation failure, the removal of sputum itself and measures to avoid fatigue of the respiratory muscles are also necessary to improve patient outcomes. Our report focused on methods to facilitate the removal of sputum. Although various methods are used to remove sputum (e.g., rib cage compression), their effectiveness has not been clarified in either experimental or clinical studies [11]. The variable effect of minitracheostomy on the rate of post-extubation respiratory failure secondary to sputum retention is influenced by multiple factors, including differing patient populations. Risk factors for extubation failure include age >65 years and chronic cardiac and lung diseases [12]. In particular, pneumonia, which is characterized by changes in sputum production, dyspnea, and cough [13], is an independent predictive variable for extubation failure [14,15]. The retention of airway secretions causes breathing difficulty and respiratory muscle fatigue [2]. Therefore, prevention of both sputum retention and respiratory muscle fatigue (e.g., one hour rest after a successful spontaneous breathing trial [16]) is necessary.

Of note, it is difficult to predict whether a patient undergoing minitracheostomy will fail extubation. Although statistical differences were not tested, SOFA scores were higher in the successful extubation group, and APACHE2 and age were higher in the reintubation group. A systematic review conducted by Torrini found that age, history of cardiac disease, history of respiratory disease, Simplified Acute Physiologic Score II score, pneumonia, duration of mechanical ventilation, heart rate, Rapid Shallow Breathing Index, negative inspiratory force, lower PaO_2_/FiO_2_ ratio, lower hemoglobin level and lower Glasgow Coma Scale before extubation were significantly associated with extubation failure [17]. In our report, the patient was an elderly pneumonia patient and critically ill with organ dysfunction, which may have contributed to the failed extubation. We believe prophylactic minitracheostomy may be useful in patients with many comorbidities and frail conditions, as they are less efficient at removing secretions. However, further analysis of association between prophylactic minitracheostomy and fail extubation cannot be performed due to lack of data.

This retrospective study had several limitations. First, there was a lack of a control group. It is unclear whether prophylactic minitracheostomy could have reduced reintubation without comparisons in similar patient populations. Second, the selection of patients at high risk of reintubation was subjective. Therefore, it was necessary to create standardized criteria for patients at high risk of reintubation. Third, we have not been able to show vital sign data before and after the minitracheostomy procedure. Therefore, we could not discuss the efficiency of the minitracheostomy. However, the results of this study showed that the immediate prophylactic use of minitracheaostomy after extubation might prevent reintubation. Further studies involving larger sample sizes and more robust study designs were warranted to assess the effectiveness of minitracheostomy for patients diagnosed with pneumonia.

## Conclusion

In summary, this case series showed that the clinical outcome of intensive care patients who underwent prophylactic minitracheostomy. Our findings suggest that the use of prophylactic minitracheostomy may protect some patients from respiratory failure after extubation. Further prospective studies in larger populations are warranted to investigate the effect of the prophylactic use of minitracheostomy.
